# A Damage-Control Strategy With Immediate Thoracic Endovascular Aortic Repair (TEVAR) for Polytrauma With Blunt Thoracic Aortic Injury and Intra-abdominal Hemorrhage: A Case Report

**DOI:** 10.7759/cureus.107868

**Published:** 2026-04-28

**Authors:** Yasutaka Saito, Shinichiro Irabu, Hirotaka Yamamoto

**Affiliations:** 1 Department of Hepatobiliary and Pancreatic Surgery / Acute Care Surgery, Seirei Hamamatsu General Hospital, Hamamatsu, JPN

**Keywords:** blunt thoracic aortic injury, damage-control strategy, polytrauma, splenic injury, thoracic endovascular aortic repair

## Abstract

We report the case of a man in his 70s who sustained polytrauma in a motor vehicle collision and presented in profound hemorrhagic shock. Focused assessment with sonography for trauma was positive for intra-abdominal fluid, and chest radiography demonstrated marked mediastinal widening. After endotracheal intubation and emergency transfusion, contrast-enhanced computed tomography revealed hemoperitoneum due to severe splenic hilar injury and blunt thoracic aortic injury with aneurysmal morphology and subadventitial hematoma, raising concern for impending rupture. Because both lesions were potentially fatal and endovascular repair required preparation time, a damage-control strategy was adopted. The patient underwent emergency laparotomy with splenectomy, distal pancreatectomy, and temporary abdominal closure for abdominal hemorrhage control, followed immediately by thoracic endovascular aortic repair. The abdomen was definitively closed on hospital day 3, and the postoperative course was uneventful. The patient was discharged home on hospital day 39. This case highlights that, in selected patients with polytrauma and unstable thoracic aortic morphology, the key issue is not only whether thoracic endovascular aortic repair is indicated, but how hemorrhage control, aortic exclusion, and resuscitation should be sequenced without avoidable delay. Incorporating thoracic endovascular aortic repair into damage-control care may be effective when supported by rapid multidisciplinary decision-making, parallel preparation, and strict hemodynamic management.

## Introduction

Blunt thoracic aortic injury (BTAI) remains one of the most lethal consequences of blunt trauma. Since the adoption of endovascular techniques, thoracic endovascular aortic repair (TEVAR) has become the preferred treatment for anatomically suitable patients who require repair, with better short-term outcomes than open repair in many series [1,2].

A contemporary grading system classifies BTAI into grade I intimal tear, grade II intramural hematoma, grade III pseudoaneurysm, and grade IV rupture [3]. However, the optimal timing of repair remains controversial. Although delayed repair with strict anti-impulse therapy is often favored in patients without signs of imminent rupture, not all patients can be safely managed with delay, particularly in the setting of severe concomitant injuries [4,5].

This issue becomes especially challenging in polytrauma, in which life-threatening hemorrhage from another source may simultaneously require damage-control surgery. In such cases, the key problem is not simply whether TEVAR is indicated, but how hemorrhage control, aortic repair, and resuscitation should be sequenced without avoidable delay. We report a patient with hemorrhagic polytrauma and BTAI with aortic morphology judged unsafe for delayed repair, in whom emergency TEVAR was performed immediately after damage-control laparotomy, and we discuss the practical implications of incorporating TEVAR into a damage-control strategy.

## Case presentation

**Figure 1:**
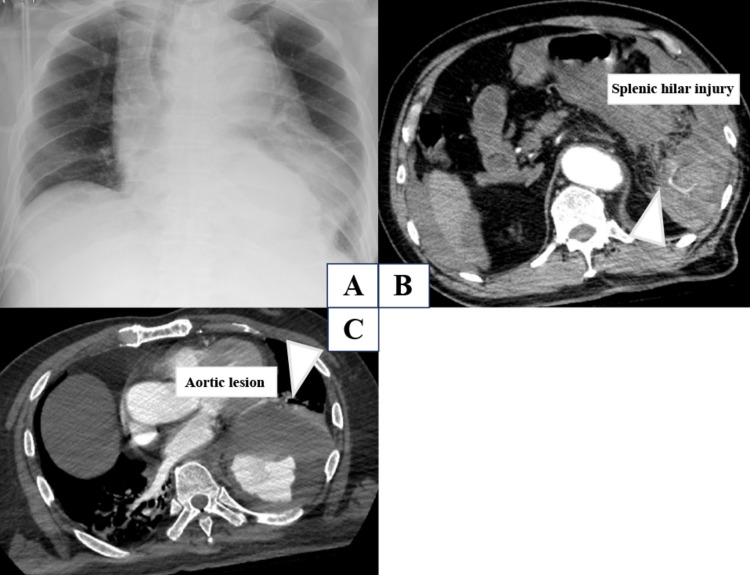
Imaging findings on admission. (A) Chest radiograph showing marked mediastinal widening. (B) Contrast-enhanced computed tomography showing splenic hilar injury with contrast extravasation (arrow) and hemoperitoneum. (C) Contrast-enhanced computed tomography demonstrating a traumatic subadventitial hematoma (arrow) in the descending thoracic aorta with aneurysmal morphology, consistent with grade II blunt thoracic aortic injury.

A man in his 70s with no known medical history was transferred to our emergency department after a motor vehicle collision. On arrival, he was in profound shock, with a heart rate of 130 beats/min and unmeasurable blood pressure. A seatbelt sign was present across the bilateral lateral chest wall. Focused assessment with sonography for trauma was positive for intraperitoneal fluid, and chest radiography showed marked mediastinal widening (Figure 1A). Because both intra-abdominal hemorrhage and thoracic aortic injury were suspected, endotracheal intubation and emergency transfusion were initiated. Although emergent laparotomy would usually precede trauma pan-scan computed tomography in a patient with profound shock and a positive FAST examination, marked mediastinal widening raised concern for concomitant thoracic aortic injury. After initiation of emergency transfusion, the patient showed a transient hemodynamic response, which allowed rapid contrast-enhanced trauma pan-scan computed tomography to be obtained under strict monitoring in order to determine treatment priority between the two competing life-threatening injuries. Resuscitative endovascular balloon occlusion of the aorta (REBOA) was not used because thoracic aortic injury was strongly suspected, and aortic balloon occlusion was considered potentially hazardous in this setting.

Computed tomography revealed hemoperitoneum with severe splenic hilar injury and contrast extravasation (Figure 1B). A laceration of the lower pole of the spleen and contusion of the pancreatic tail were also identified. In addition, the descending thoracic aorta showed aneurysmal morphology with a traumatic subadventitial hematoma (Figure 1C). Although this aneurysmal change had not been previously documented, we clinically considered it likely to represent a pre-existing aneurysmal lesion complicated by traumatic injury. The patient, therefore, sustained a high-grade splenic injury involving the splenic hilum with active hemorrhage, corresponding to AAST-OIS grade IV, an associated sternal fracture, and grade II BTAI of the descending thoracic aorta with subadventitial hematoma and aneurysmal morphology. The Injury Severity Score was 32, the Revised Trauma Score was 4.21, and the predicted probability of survival by TRISS was 0.18 [6-8].

Because immediate abdominal hemorrhage control was required, but the thoracic aortic lesion was considered too unstable for observation during prolonged resuscitation, a hybrid damage-control strategy was selected. The patient was transferred directly from the computed tomography suite to the operating room. Emergency laparotomy was performed first. A large amount of intraperitoneal blood was encountered. The lower pole of the spleen was lacerated, and the splenic hilum was severely disrupted. Because the pancreatic tail was also contused, distal pancreatectomy and splenectomy were performed. The splenic vessels were divided en bloc using a stapling device, and temporary abdominal closure was completed as part of damage-control surgery. The laparotomy phase was completed in 63 minutes, with an estimated blood loss of 3,950 mL.

**Figure 2:**
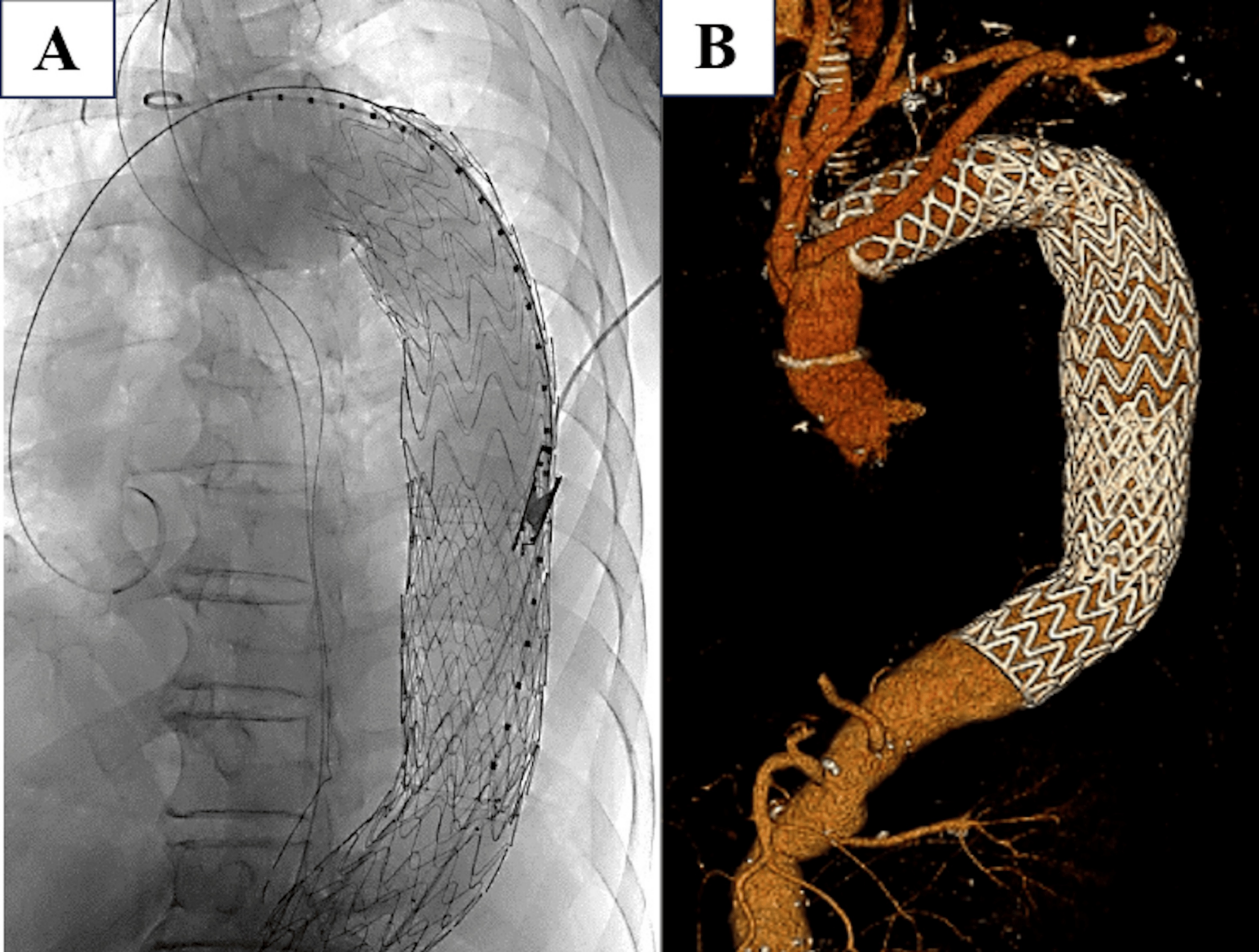
Endovascular and postoperative imaging findings. (A) Fluoroscopic image obtained during emergency thoracic endovascular aortic repair showing stent graft deployment across the injured thoracic aortic segment. (B) Three-dimensional reconstructed image after subsequent total arch replacement and additional thoracic endovascular aortic repair.

While the abdominal procedure was ongoing, the endovascular team prepared the stent graft and completed the necessary measurements to minimize delay before aortic repair. Immediately after temporary abdominal closure, the patient underwent emergency thoracic endovascular aortic repair without systemic heparinization because of the ongoing hemorrhagic risk from the associated injuries. Two cTAG stent grafts (Gore; TGM404015J and TGMR454520J) were deployed in an overlapping fashion to exclude the injured thoracic aortic segment. Fluoroscopy demonstrated successful stent graft deployment across the injured thoracic aortic segment (Figure 2A). No type Ia endoleak remained after deployment, and a small distal contrast leak was treated with balloon molding. The endovascular procedure was completed in 72 minutes, with an estimated blood loss of 30 mL, and the patient was subsequently admitted to the intensive care unit.

Definitive abdominal closure was performed on hospital day 3. Thereafter, the patient showed steady recovery without major complications and was discharged home on hospital day 39. Six months after discharge, he underwent total arch replacement and additional thoracic endovascular aortic repair for the aortic arch aneurysmal lesion (Figure 2B).

## Discussion

**Figure 3:**
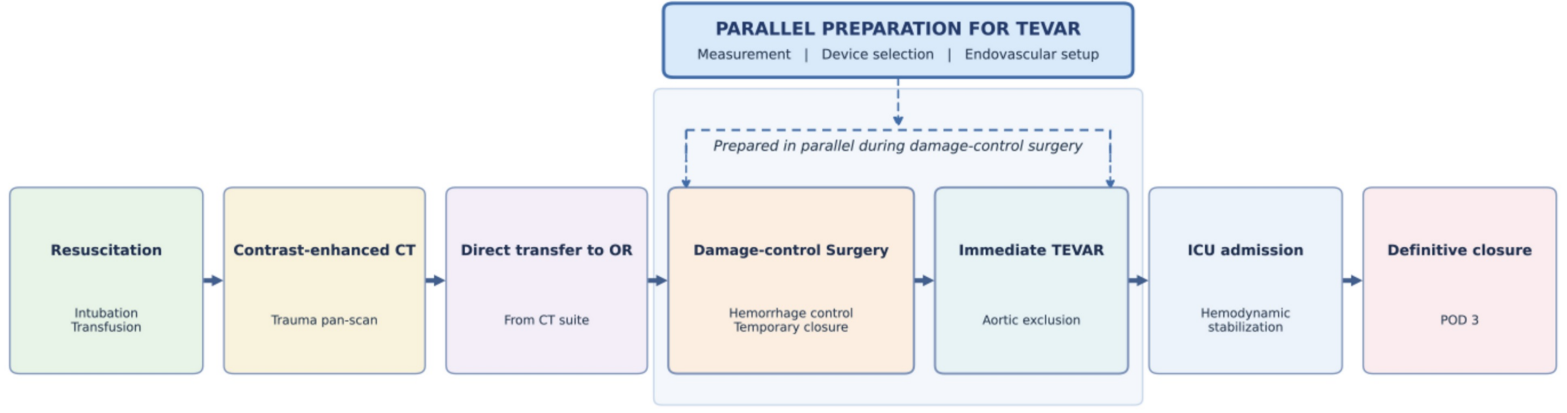
Acute-phase sequencing of damage-control strategy in the present case. Damage-control surgery was performed first for intra-abdominal hemorrhage, while preparation for TEVAR proceeded in parallel. Emergency TEVAR was performed immediately after temporary closure, followed by ICU admission and definitive abdominal closure on postoperative day 3. This schematic figure was created by the authors using the Python Matplotlib library.

The acute-phase sequencing of the damage-control strategy in this case is summarized in Figure 3. This case is best understood not simply as a question of whether TEVAR was indicated, but as one of treatment sequencing within a damage-control strategy. The traumatic lesion itself was classified as grade II BTAI because the acute finding was subadventitial hematoma. This case did not represent spontaneous type B aortic dissection; rather, management was influenced by the combination of traumatic subadventitial hematoma, aneurysmal aortic morphology likely predating the trauma, and profound hemorrhagic shock from concomitant intra-abdominal bleeding. Because the hematoma developed in a descending thoracic aorta with aneurysmal morphology, we considered the overall lesion rupture-concerning in the setting of profound shock and marked mediastinal widening. Thus, the rationale for same-day TEVAR was based not on the injury grade alone, but on the combination of traumatic grade II injury, aneurysmal aortic morphology, and physiologic instability. The novelty of this case lies not in the indication for urgent TEVAR itself, but in the commander-led sequencing of contrast-enhanced CT, damage-control surgery, parallel endovascular preparation, and immediate TEVAR in a patient with competing life-threatening injuries.

Current literature generally supports TEVAR as the preferred repair strategy for anatomically suitable BTAI. However, the optimal timing of repair remains controversial. The 2011 Society for Vascular Surgery guideline recommended endovascular repair for grade II to IV injuries when feasible [1], whereas the 2015 EAST practice management guideline suggested delayed repair for many patients, provided that effective anti-impulse therapy can be maintained [4]. The 2022 ACC/AHA guideline further states that aortic intervention is reasonable for grade 2 blunt traumatic thoracic aortic injury with high-risk imaging features, whereas nonoperative management may be reasonable for grade 2 injury without such features. In addition, from an aortic disease perspective, the same guideline identifies maximal aortic diameter >47-50 mm as a high-risk imaging feature for type B intramural hematoma [9]. Although this framework does not directly define traumatic injury, it supports our concern that delayed observation would be unsafe in this case, given the aneurysmal morphology and the maximal aortic diameter of 94 mm. Registry- and multicenter-based studies in the TEVAR era have further suggested that delayed repair may be associated with lower mortality in patients without signs of imminent rupture [10]. A more recent propensity score-matched analysis also supported this tendency [11]. Another recent analysis reported that, compared with urgent or emergent TEVAR, elective TEVAR was associated with lower postoperative stroke and pulmonary complications, while perioperative and 5-year mortality were similar [12].

Our case should therefore be interpreted as a selective deviation from the conventional damage-control sequence, justified by the need to characterize a suspected concomitant thoracic aortic lesion before proceeding with hemorrhage control. It is best viewed as an exception defined by competing life-threatening injuries and physiologic instability, rather than as a proposed routine pathway for all unstable trauma patients. We do not argue that all grade II or grade III BTAI in polytrauma require the same-day TEVAR. Rather, this case suggests that selected patients with competing hemorrhagic priorities and rupture-concerning aortic morphology may benefit from an individualized hybrid strategy. In our patient, immediate laparotomy was necessary for control of splenic hilar bleeding, but prolonged resuscitation in the intensive care unit before aortic repair was considered unsafe. The treatment sequence was therefore designed to achieve rapid abdominal hemorrhage control first, followed immediately by definitive aortic exclusion before further physiologic deterioration could occur.

This case also highlights several practical pitfalls when incorporating TEVAR into a damage-control strategy. Unlike standard damage-control procedures, TEVAR can introduce hidden delays related to imaging review, device measurements, graft selection, inventory retrieval, fluoroscopic setup, room availability, and coordination between trauma, anesthesia, and endovascular teams. In polytrauma, these delays matter because prolonged time to aortic exclusion may offset the theoretical advantages of endovascular repair, especially if blood pressure control, transfusion strategy, and coagulation management become fragmented during the transition between procedures. Accordingly, successful use of TEVAR in damage-control care depends not only on endovascular feasibility but also on minimizing procedural dead time.

For that reason, the organizational component of this case is as important as the technical one. In our institution, the commander-centered decision-making process allowed the operative sequence to be shared early among all involved teams, including the need to avoid REBOA, proceed directly from the CT suite to the operating room, perform damage-control laparotomy first, and prepare TEVAR in parallel. This parallel preparation minimized the interval between temporary abdominal closure and endovascular repair and helped maintain continuity in hemodynamic goals and transfusion management. The lesson from this case is not merely that emergency TEVAR can be performed after laparotomy, but that it should be attempted only within a coordinated command-and-control framework that explicitly addresses timing, task allocation, and communication.

Taken together, this case demonstrates that the value of TEVAR in polytrauma lies not only in its minimally invasive nature, but in how effectively it can be integrated into an overall resuscitative strategy. When multiple lethal injuries coexist and delayed aortic repair is judged unsafe, a commander-led hybrid approach consisting of damage-control laparotomy followed by immediate TEVAR may provide a feasible approach in selected settings.

## Conclusions

In polytrauma with competing hemorrhagic priorities, the key issue is not only whether TEVAR is indicated, but how hemorrhage control, aortic exclusion, and resuscitation can be sequenced without avoidable delay. This case suggests that, in selected patients in whom delayed aortic repair is judged unsafe, a commander-led hybrid damage-control strategy consisting of immediate laparotomy followed by TEVAR may provide a feasible treatment approach.
